# Passive retention of simulated larvae on coral reefs

**DOI:** 10.1098/rsos.241708

**Published:** 2025-05-23

**Authors:** Jim Greenwood, C. J. Sun, Christopher Doropoulos, Damian Thomson, Mark Baird, J. Porobic, Scott Condie

**Affiliations:** ^1^ CSIRO Environment, Perth, Western Australia, Australia; ^2^ CSIRO Environment, Brisbane, Queensland, Australia; ^3^ CSIRO Environment, Hobart, Tasmania, Australia

**Keywords:** coral larvae, dispersal, local retention, ocean model

## Abstract

The extent to which local coral populations are self-sustaining through local recruitment has important implications for managing coral reef systems. However, a lack of understanding has led to overly simplistic representation of this phenomenon in coral reef population models. In this study, we simulate the dispersal of artificial larvae from 24 selected individual reefs across the Great Barrier Reef, Australia, over a spawning period in December 2016, to identify key physical factors influencing their retention. We found the dispersal pattern of larvae differed depending on whether they are well mixed throughout the water column and transported by depth-averaged velocity or floating near the surface, with well-mixed populations following more circuitous routes and dispersing more slowly. Retention time (*R_t_*) varies widely between reefs, with most of the variation observed in this study (*r*^2^ = 0.90) explained by reef area (*A*) represented by the empirical power law relationship *R_t_* = 10.34 A^0.65^, or alternatively by a combination of reef area and mean water depth ($\bar{h}$) using the linear relationship *R_t_* = 1.23(*A*) – 6.38($\bar{h}$). The formation of tidal eddies and being situated among closely aggregated reefs are shown to be important factors for larval retention. Simple retention relationships like these have the potential to be incorporated into larval connectivity modelling and reef meta-community modelling where reef area and water depth are known. Further research is needed to determine how different oceanographic conditions and interannual variability will affect these relationships.

## 1. Introduction

Since the 1950s, global coverage of living coral has halved owing to the cumulative impacts of global warming, overfishing, habitat loss, pollution and disease [1]. The decline has fuelled efforts to protect remaining coral reefs and develop management strategies that enhance ecosystem resilience. The resilience of coral populations and their ability to recover from bleaching and other disturbances is highly dependent on patterns of larval connectivity (e.g. [2–6]). Understanding the extent to which local coral populations are self-sustaining through local recruitment, as opposed to relying on larvae from distant reefs, is crucial for managing coral reefs and aiding their adaptation to changing conditions, such as increased heat tolerance. This knowledge is vital for the prioritization of reefs for differing conservation strategies, such as designating marine reserve boundaries or selecting reefs for enhanced protection or restoration [7–10]. For example, when local recruitment is high and inter-reef connectivity is low, protective measures will be more effective locally with reduced separation needed to connect protected areas. This may be increasingly important as warming oceans shorten larval dispersal phases and increase the proportion of larvae retained on natal reefs [11].

Although coral larvae have the potential for long-distance dispersal, genetic studies and field surveys indicate a substantial level of local coral recruitment [12–15]. Coral larvae have very limited motility [16] beyond adjusting their vertical position in the water column [17]. Therefore, local retention is mainly determined by environmental conditions such as recirculating flows and flushing rates ([15,18–22], and biological factors such as the age at which larvae become competent to locate and settle on suitable substrate [23–26]. In this study, we focus on the role that physical retention plays in determining local recruitment of a broadcast-spawning coral.

Given the small size (<800 µm) and challenges associated with *in situ* tracking of coral larvae, our understanding of their passive retention largely stems from models of oceanic dispersal (e.g. [20,22,27]). Early work by Black *et al*. [22] modelled particle dispersal from artificial reefs of different idealized shapes and sizes on the Great Barrier Reef (GBR) and derived a simple equation relating flushing rate to reef size, water depth and depth-averaged flow on the reef. Their equation suggests that particle concentrations on and around the reef decay exponentially over time, with retention times tending to increase with reef size. Retention time was particularly sensitive to the dimension of the reef perpendicular to the current, which has an important bearing on the formation of eddies in the lee of the reef [28]. Subsequent studies in the southern and central GBR have shown that local retention varies with tidal and mean currents, local bathymetry and reef density [20]. These results highlight that the surrounding seascape can affect individual reef retention properties.

The circulation around reefs is affected by complex bathymetry and small-scale flow features such as nonlinear waves and eddies. Resolving such spatial features in numerical circulation models has been shown to increase predicted rates of local retention [27]. Consequently, coarser regional-scale circulation models consistently underestimate retention rates. These errors can be carried forward into reef meta-population models and potentially have a major impact on their ecological predictions (e.g. [4,29,30]). To avoid this, some meta-community models assume a constant rate of local recruitment [7,29,31,32]. For example, in their connectivity-forced coral ecosystem model, Bozec *et al*. [29] set a minimum local recruitment rate of 28% for all GBR reefs based on a field experiment at Helix reef [18], and mortality and competency curves derived from Connolly & Baird [33]. While the limitations of using uniform retention rates across all reefs are broadly recognized, there has been insufficient information to derive more realistic relationships.

Broadcast-spawning corals release propagules that typically begin as passive, positively buoyant particles [34,35]. As larvae develop, they lose buoyancy [36] and gradually sink over time [37]. This downward trajectory is potentially enhanced by active migration towards the seabed in response to biophysical settlement cues [17,24] or opposed by photomovement towards the surface [38]. Regardless, enhanced and variable vertical turbulent mixing associated with advective flow over coral reefs [39] may rapidly overcome any vertical structure in the distribution of larvae [40]. Changes in vertical distribution can significantly affect larval dispersal patterns owing to vertical shear in the hydrodynamic flow (e.g. [41]) and must be considered in estimating dispersal of larvae using circulation models. Although three-dimensional particle-tracking simulations offer a potential solution to this problem (e.g. [42,43], lack of *in situ* knowledge about changes in the vertical position of coral larvae over time and the increased computer resources required for three-dimensional particle-tracking remain problematic. To simplify the problem, depth-averaged flow conditions [20,22,27] or fixed-depth dispersal (e.g. [4,44,45]) have commonly been employed, although the broader consequences of doing so have not been quantified.

The overall aim of the present study is to compare the passive retention of particles from a selection of reefs of different sizes and locations across the GBR, to inform the parameterization of local coral recruitment in regional-scale meta-population models of the GBR. This approach assumes that the horizontal grid resolution of a hydrodynamic model used to identify GBR-scale inter-reef connectivity for a meta-population study will be insufficient to also accurately predict local retention of passive particles needed to estimate local recruitment. In this case, local recruitment must be parameterized separately into the meta-population model. We present a potential improvement to the constant rate parameterization used in previous studies of the GBR [29,32] that depends on reef area and mean water depth. We approach this problem by conducting fine grid resolution particle-tracking experiments for 24 selected GBR reefs of diverse size and morphology to identify key reef features and physical mechanisms associated with high retention. We also compare results using surface and depth-averaged velocities to highlight the importance of understanding variations in the vertical distribution of larvae.

## 2. Methods

**Figure 1.**
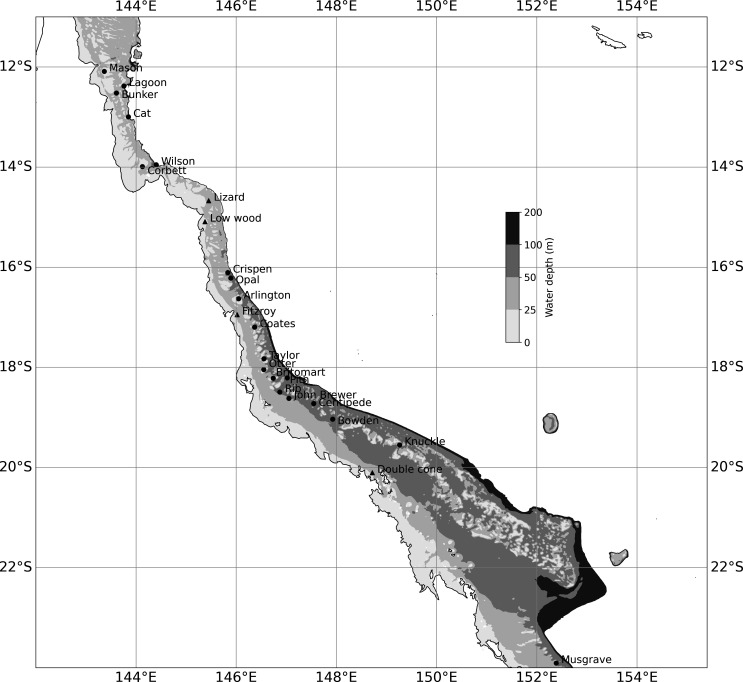
Map showing the location of reefs in the GBR where modelled particle dispersal experiments were conducted. Circular markers indicate submerged reefs, and triangular markers indicate island reefs (Lizard, Low Wood, Fitzroy, Double Cone). Grey filled contours show the bathymetry of the coarse-scale ‘parent’ model (within which each high-resolution reef model is nested) out to a maximum seabed depth of 200 m. The parent model has a horizontal resolution of approximately 1 km and 48 vertical layers, with 1 m resolution at the surface.

A set of numerical model experiments were conducted to investigate the passive dispersal of neutrally buoyant particles representing broadcast-spawning coral larvae, such as *Acropora* spp., that either floated near the ocean surface or were mixed throughout the water column. The same experimental set-up was applied to 24 reefs along the GBR (figure 1), varying in their size, shape, water depth and latitude. The particle-tracking experiments were conducted with Ocean Parcels [46] using a fourth-order Runge–Kutta integration scheme. Use of a fine spatial grid resolved more of the key flow structures contributing to retention, thereby more accurately representing the advective transport that tends to dominate dispersal on reef systems [22]. This removed the need for additional horizontal mixing based on stochastic parameterizations such as adding a random walk to particle movements.

The advective field for each reef was determined from previously archived hourly output of either surface or depth-averaged velocity estimated by a high-resolution three-dimensional hydrodynamic model nested within a broader regional hydrodynamic model. The model and the nesting approach are part of the eReefs project that simulates the environmental conditions of the GBR at multiple scales [47,48]. The hydrodynamic models are configurations of the Sparse Hydrodynamic Modelling Code that contains boundary conditions developed specifically for high-resolution nesting [49,50] and are detailed in two previous publications that used outputs from the same archived reef simulations for different purposes [51,52]. An approximately 1 km resolution grid was used as the standard ‘parent’ model for all reefs, with finer resolution models nested at each of the 24 reefs. Grid resolution and associated parameters for each nested reef model are provided in the electronic supplementary material, table S1. Note that the eReefs models are configured on a staggered Arakawa C grid, whereas here we use output that has been re-gridded onto an Arakawa A grid to obtain both velocity components at the centre of each grid.

**Figure 2.**
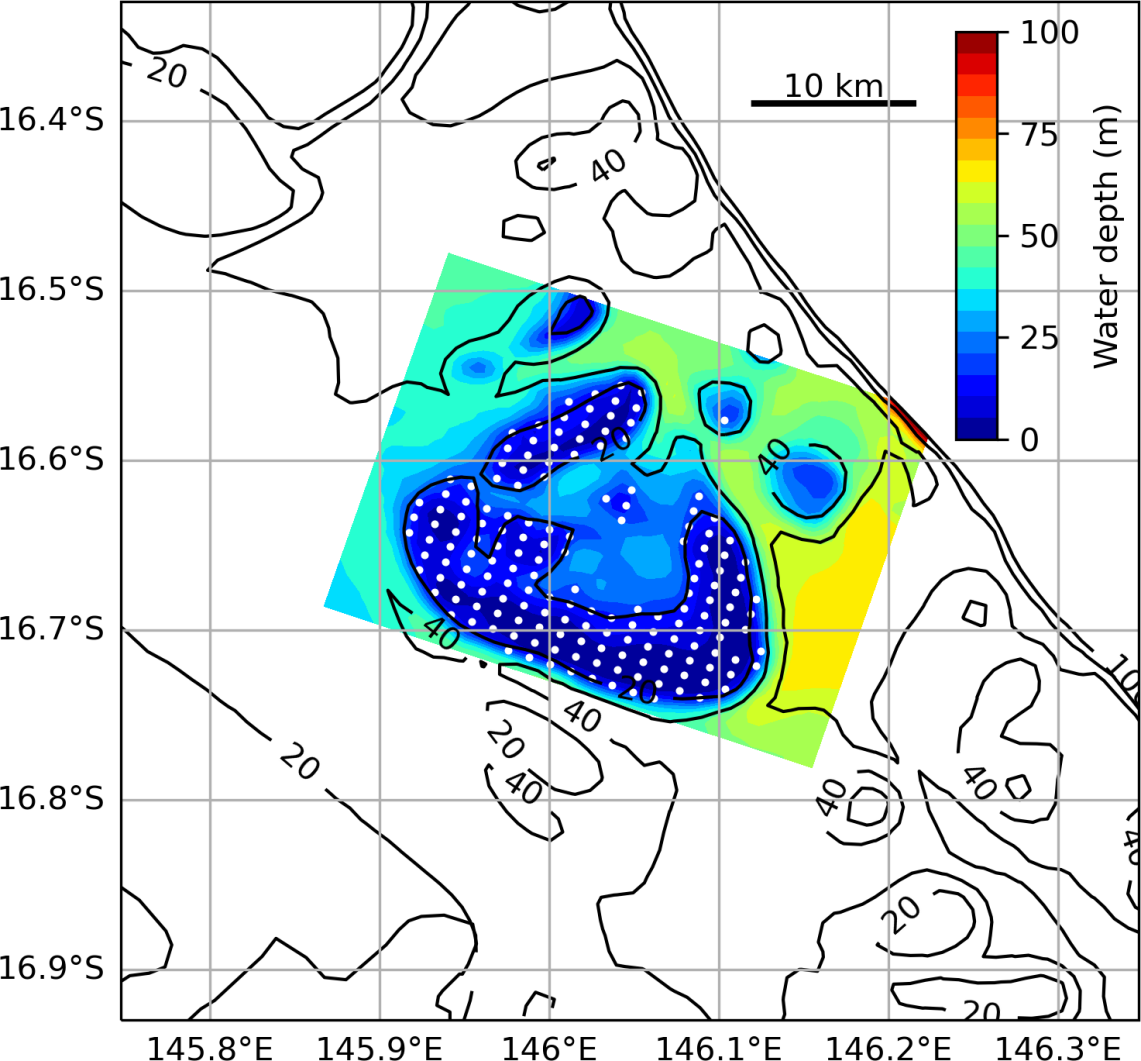
Example from Arlington reef of the distribution of initial particle positions used in the dispersal experiments (white dots; only every other release point (fourfold reduction) is shown here to avoid obscuring the underlying bathymetry). Filled contours show bathymetry across the fine-scale reef model domain every 5 m and black line contours show bathymetry from part of the ‘parent’ model at 20, 40, 60, 80 and 100 m depth.

Each reef was defined by one or more 20 m closed depth contours on the nested model grid and confirmed by visual inspection. Reef area, *A*, was then calculated as the summed area of the grid cells with a seabed depth of 20 m or less (see the electronic supplementary material, table S1). Particle releases were initiated at the centre of each of these reef area grid cells. As an example, the reef area and particle release points for Arlington reef, defined by 642 separate grid cells, are shown in figure 2. While this definition of reef area may misrepresent the actual area of coral habitat on each reef, it captures the areas of highest coral abundance and provides a standard way to initialize particles and compare reefs. We note, for later discussion, that the reef areas defined in this way are generally smaller than the management polygons used elsewhere to define reefs of the GBR (e.g. [29,45]), and smaller than the reef region defined by Black *et al*. [22] in their study of dispersal from GBR reefs. To allow later comparison between results obtained using the nested models with those using only the parent model (coarser horizontal resolution), the initial particle positions on the fine grid were also transferred directly to the coarse grid. For some of the longer simulations conducted on the larger reefs, particle density was reduced evenly across the reef to reduce compute time. Additional experiments indicated model results were not unduly affected by these reductions in particle density. This is exemplified later for Arlington reef (see figure 6d).

In each experiment, one particle was released simultaneously from the centre of each of the specified model grid cells once every 6 h for 24 h, resulting in a total of five separate releases (e.g. a total of 3210 particles were released from Arlington reef). The decision to release particles every 6 h was a practical one to reduce overall particle numbers. In additional experiments, we found that releasing particles every hour did not unduly affect the result (exemplified later in figure 6d). Releases started at midnight on the 20 December 2016, 6 days after the full moon, corresponding to the end of the typical lunar-induced coral spawning period (four to seven nights: [53]) and consistent with observations of coral spawning in the northern GBR reefs during 2016 [45]. To ensure consistent comparisons, the same spawning period was used for all 24 reefs, while acknowledging that the timing of coral spawning varies with species and latitude.

The trajectory of the particles within the modelled velocity field was estimated by calculating their position every 20 min for a minimum of 5 days up to a maximum of 20 days. Analysis of the particle trajectories was made using hourly output. Particles are described as being ‘over the reef’ while they remain within the 20 m depth contour(s) specifically used to define the reef area and are considered to have moved ‘off the reef’ once they cross this contour. In this study, retention refers to particles which remain over the reef, and we define total retention time, *R_t_*, as the time it takes to reduce the number of particles over the reef to 5% of the number released. In cases where particle numbers over the reef drop below 5% and then recover before being lost completely, we take *R_t_* as the last time particle numbers over the reef were at the 5% level. It is important to note that elsewhere the retention of particles in association with coral reefs refers to a larger domain that includes areas of deeper water surrounding the reef where particles may be temporarily retained before returning to the shallower reef area [22].

For the four island reefs (figure 1), an artificial velocity field of magnitude 1 × 10^–6^ m s^−1^ acting perpendicular to the land was added in each of the coastal cells to avoid particles becoming stranded. The method for this was adapted from Kaandorp *et al*. [54] taking account of any grid rotation. This is a common particle-tracking problem when using velocity fields configured on an Arakawa A grid because both velocity components tend towards zero in the same way as particles approach a land cell.

## 3. Results

**Figure 3.**
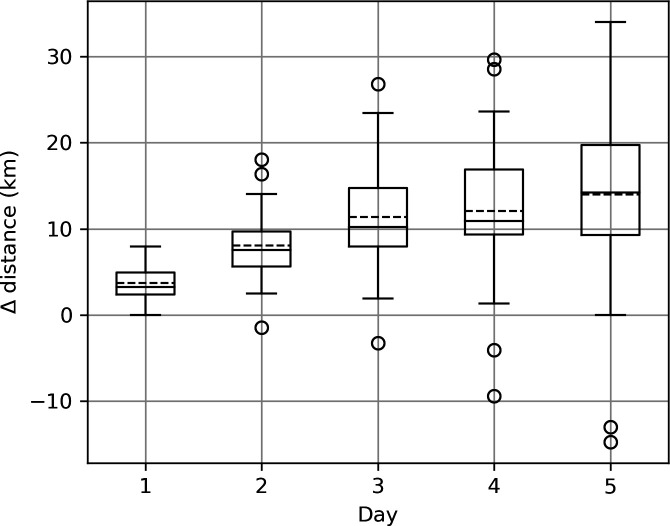
Difference in cumulative distance (km) travelled over 1–5 days (average of all particles and all reefs) subjected to either surface or depth-averaged velocity. The values were calculated as the distance travelled under surface velocity conditions minus the distance travelled under depth-averaged velocity conditions. In each box-whisker plot, the box extends from the first quartile to the third quartile of the data, with a solid line at the median and a dashed line at the mean. The whiskers extend from the box to the furthest point lying within 1.5 times the interquartile range from the box. Flier points are those past the end of the whiskers. Note that the mean distance travelled under depth-averaged flow conditions exceeded that under surface flow conditions for only two reefs indicated by the negative values of the flier points.

**Figure 4.**
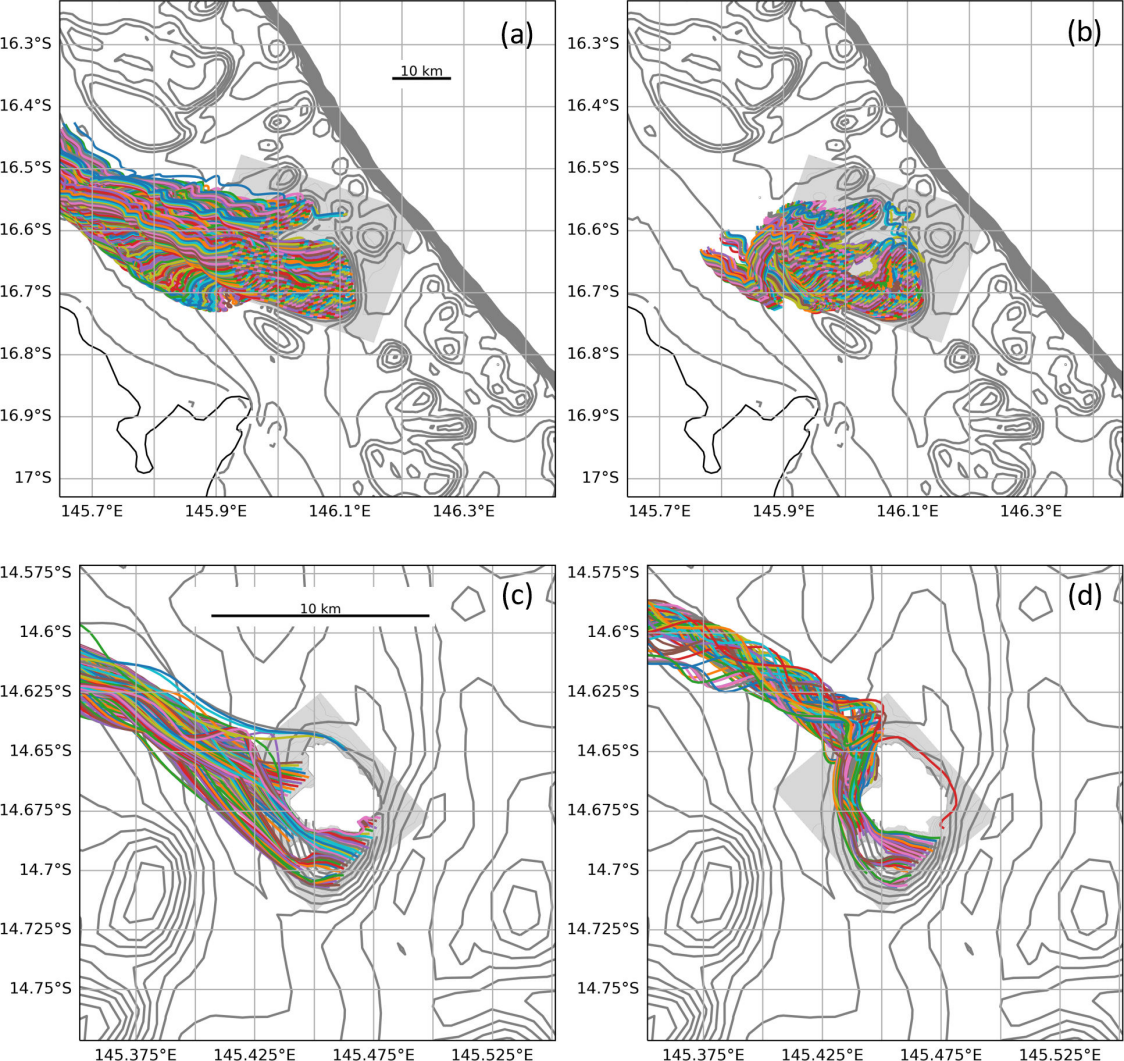
Comparison of 5 day particle trajectories for Arlington reef (top) and Lizard Island (bottom) resulting from (a) and (c) surface, or (b) and (d) depth-averaged advection. Bathymetry from the 1 km ‘parent’ model is shown by the grey contours, and grey shading defines the fine-resolution nested model grid domain. The particle trajectories have been cut off at 145.65° E in (a), and at 145.350° E in (c) and (d) to provide more detail around the nested grid. In (c) and (d), the island topography has been masked in white.

Analysis of particle trajectories in high-resolution models of 24 reefs across the GBR revealed some important trends. Particle trajectories varied depending on whether surface or depth-averaged velocities were used. Daily surface transport was typically higher than depth-averaged transport (figure 3) because of more direct exposure to wind-driven surface currents and less direct exposure to bottom drag. Over shallow areas of reef, surface particles also tend to follow relatively straight trajectories, whereas depth-averaged currents often carried particles on more circuitous pathways through the reef matrix with periodic tidal reversals (figure 4a,b). These differences tend to disappear around islands where all trajectories are restricted by the shoreline (figure 4c,d).

**Figure 5.**
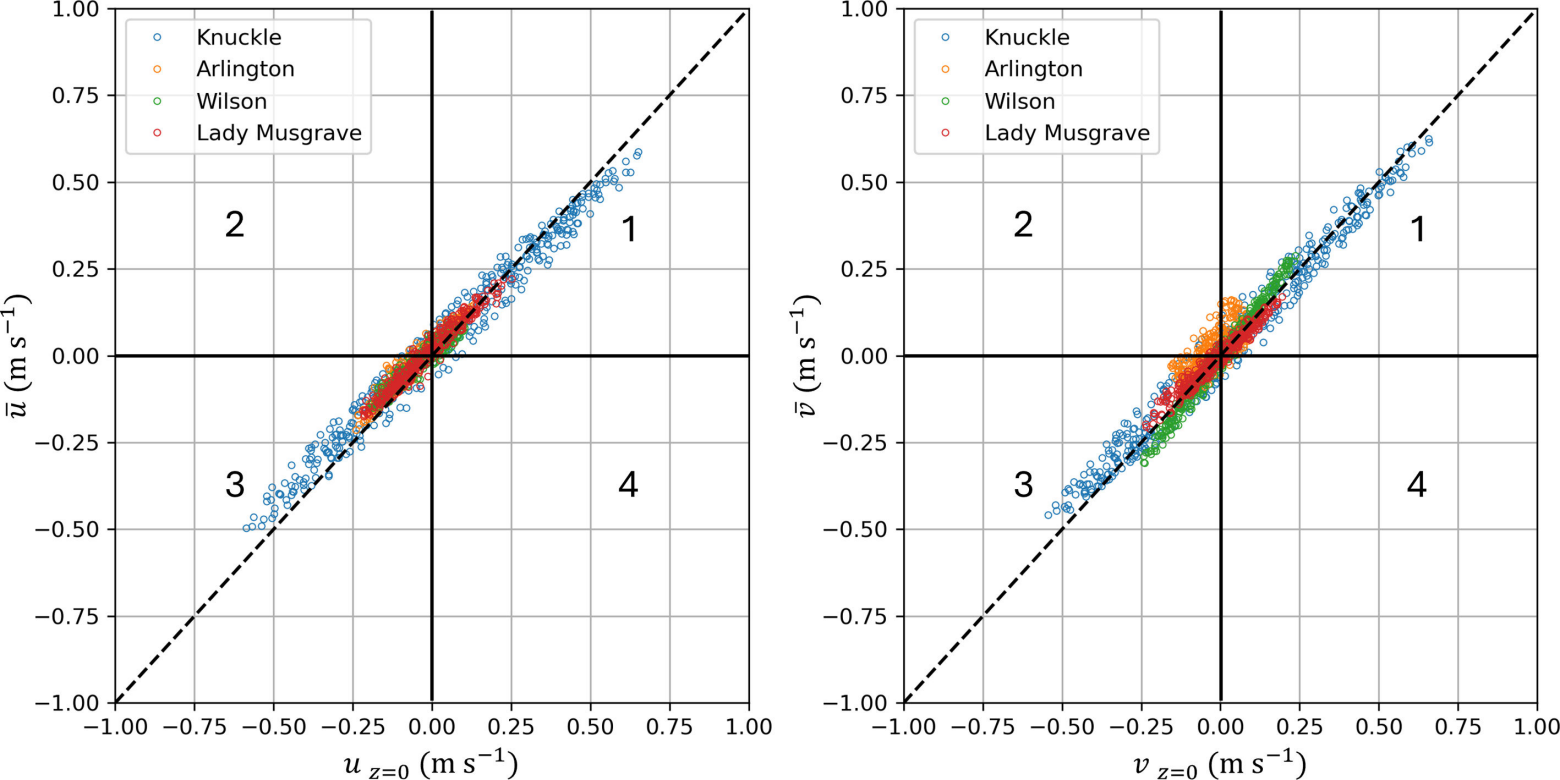
Comparison of hourly depth-averaged velocity vectors ($\bar{u},\;\bar{v}$) (m s^-1^) with hourly surface velocity vectors (*u*_*z* = 0_, *v*_*z* = 0_) (m s^−1^) for four example reefs over a period of 14 days. Positive values of *u* and *v* indicate flow to the east and north, respectively. The dashed line represents a 1 : 1 relationship between surface and depth-averaged values, and the solid black lines and numbers (1–4) define the four Cartesian quadrants referred to in the text.

Closer examination of the hourly surface and depth-averaged velocity components (in water depths <20 m) for a selection of four submerged reefs shows that their relationship is not linear and varies between reefs over the course of a full lunar tidal cycle (i.e. 14 days; figure 5). These four reefs (Wilson, Arlington, Knuckle and Lady Musgrave) cover most of the range in latitude and reef area (see figure 1 for location and the electronic supplementary material, table S1, for reef area). Variation between reefs could be partially explained by water depth. For example, Lady Musgrave reef is the shallowest of the four examples ($\bar{h}$ = 6.43 m) and displays the closest relationship between surface and depth-averaged velocity (both components), while Knuckle reef is the deepest ($\bar{h}$ = 11.39 m) and shows greater variation especially under strong flow conditions. The other two reefs, Arlington ($\bar{h}$ = 9.27 m) and Wilson ($\bar{h}$ = 9.00 m), lie somewhere between these two (figure 5). In all cases, depth-averaged velocities can exceed surface velocities at times during the two-week tidal cycle (points above the 1 : 1 line in the first Cartesian quadrant and below the 1 : 1 line in the third Cartesian quadrant in figure 5). Surface and depth-averaged flow can even be in opposite directions (points within the second and fourth Cartesian quadrants in figure 5). This can occur when the prevailing wind, which preferentially influences the surface flow, acts in the opposite direction to the tidally driven flow. The corresponding time-averaged vertical profiles of the velocity vectors at each grid point inside the 20 m depth contour (electronic supplementary material, figure S1) show some of the vertical structure in the flow that leads to this result with either sub-surface maxima or reversal in the sign of the velocity component with depth. Also evident in the electronic supplementary material, figure S1, is that the vertical structure of the flow varies spatially across the reef. Overall, there is often a complex pattern of three-dimensional flow in time and space over the course of a lunar tidal cycle that is likely to be different for each reef.

The varying difference between surface and depth-averaged flow identified above explains the mismatch in displacement distance (figure 3) and particle trajectories (figure 4) and why some reefs compare better than others. Expected variation in the vertical distribution of real larvae in time and space has encouraged us to favour the use of depth-averaged velocities for the purpose of this study.

**Figure 6.**
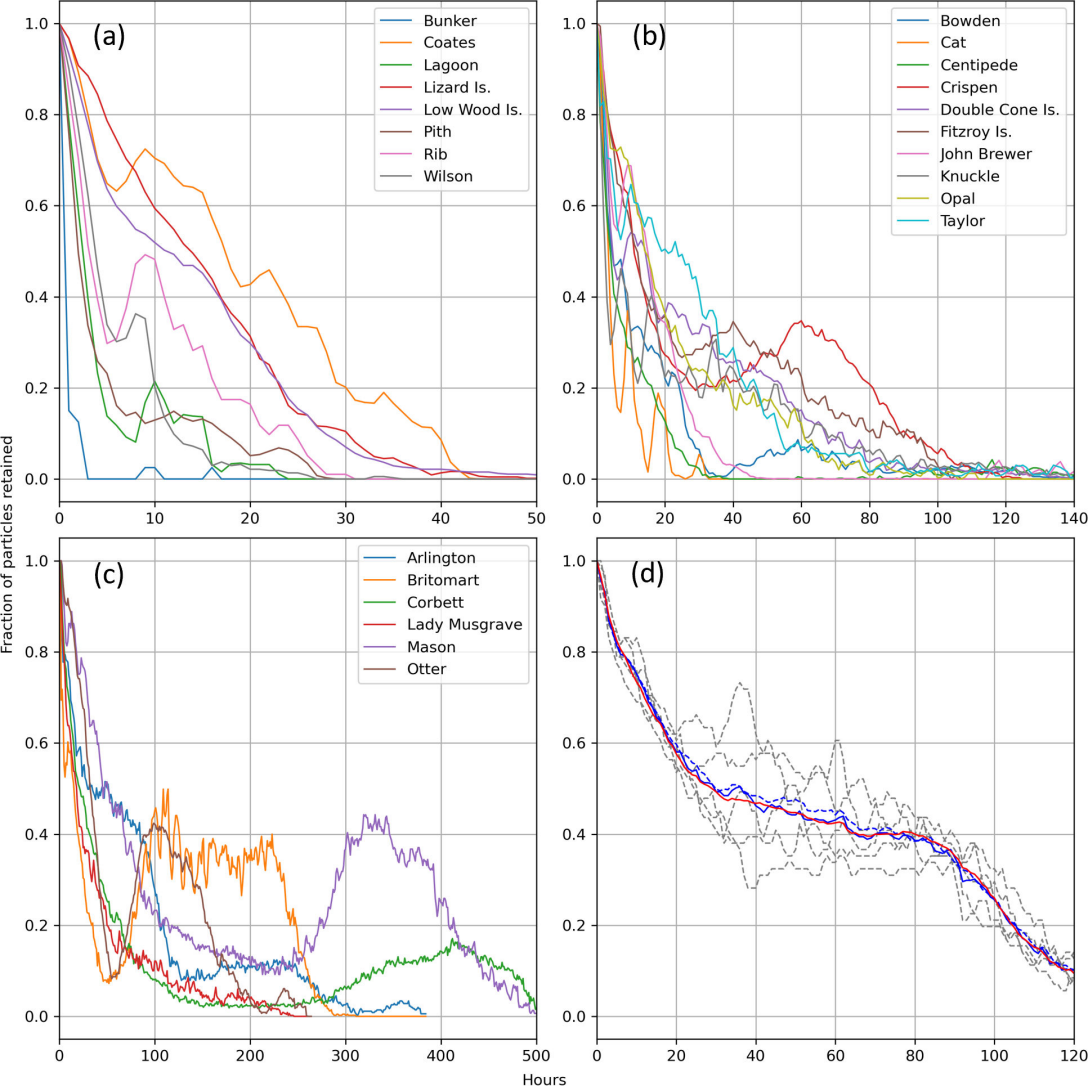
Variation in the fraction of particles retained over reefs as a function of time when driven by depth-averaged velocities. Results have been separated into those with (a) short (<50 h), (b) medium (50–150 h), and (c) long (>150 h) total retention times based on surface velocities. Panel (d) shows the temporally varying and mean particle retention for Arlington reef over the first 5 days (120 h) based on depth-averaged velocities (note that there is a partial recovery of particle concentration after 120 h; see the blue line for Arlington reef in (c)). The dashed lines in (d) show retention of particles initialised at *t* = 0, 6, 12, 18 and 24 h, where *t* = 0 is midnight on 20 December 2016, and the solid blue line is the average of these five releases. Also shown in (d) is the mean particle retention obtained for Arlington reef when the density of particle release sites is reduced ninefold (dashed blue line) to ease computational demands for runs longer than 5 days and the smoother mean particle retention obtained when particles are released hourly, rather than 6-hourly, over the first 24 h (red solid line).

The patterns of depth-averaged flow ultimately determine how long particles are retained over the reef. As particles move away from the reef, the fraction of total particles retained decreases and can be displayed as a graph of the fraction of particles retained versus time. Figure 6a–c shows the mean retention versus time curves that result from the sequential release of a set of particles at each grid point over the reef once every 6 h for 24 h (a total of five particle releases). It is important to note that this represents a temporal mean of retention time over 24 h and effectively smooths some of the variation in retention caused by the diurnal or semi-diurnal reversing tidal flow which acts to move particles on and off the reef, causing oscillations in the number of particles retained over time. These oscillations are especially noticeable when using depth-averaged velocity owing to the increased influence of tidal flow over surface wind forcing. As an example, this oscillation effect is revealed for Arlington reef over the first 5 days (120 h) of dispersal by plotting separate retention versus time curves for each of the five cohorts of particles released 6 h apart (figure 6d).

For Arlington reef, the tidal oscillations tend to be out of phase with each other and almost cancel out when averaged over 24 h, resulting in near-monotonic decay in retention (figure 6c,d). However, on other reefs (e.g. Crispen, Otter, Britomart, Arlington, Corbett and Mason), particle numbers exhibit an initial loss followed by a partial recovery (see figure 6b,c). This recycling of particles effectively extends the overall estimated retention time.

**Figure 7.**
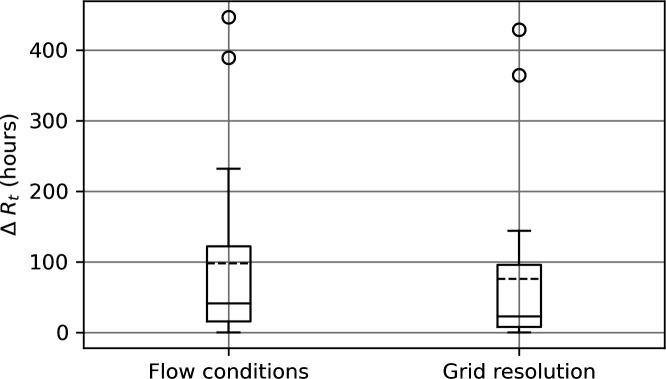
Increase in particle retention time with use of depth-averaged (rather than surface) flow conditions (left box) and enhanced model grid resolution (right box) for the submerged reefs (*n* = 20). In each case, the box extends from the first quartile to the third quartile of the data, with a solid line at the median and a dashed line at the mean. The whiskers extend from the box to the furthest point lying within 1.5 times the interquartile range from the box. Flier points are those past the end of the whiskers.

As expected from examination of particle trajectories (figure 4), retention time varies depending on whether surface velocities or depth-averaged velocities are applied. Ignoring outliers, retention times increase by 0−8 days (average approx. 4 days) for submerged reefs (figure 7). The sensitivity of retention time to velocity field depth is comparable to the sensitivity to velocity field resolution, where retention times increased by 0−6 days (average approx. 3 days) when resolution was enhanced from the ‘parent’ model (approx. 1000 m) to the ‘nested’ models (approx. 200 m; electronic supplementary material, table S1) (figure 7).

**Figure 8.**
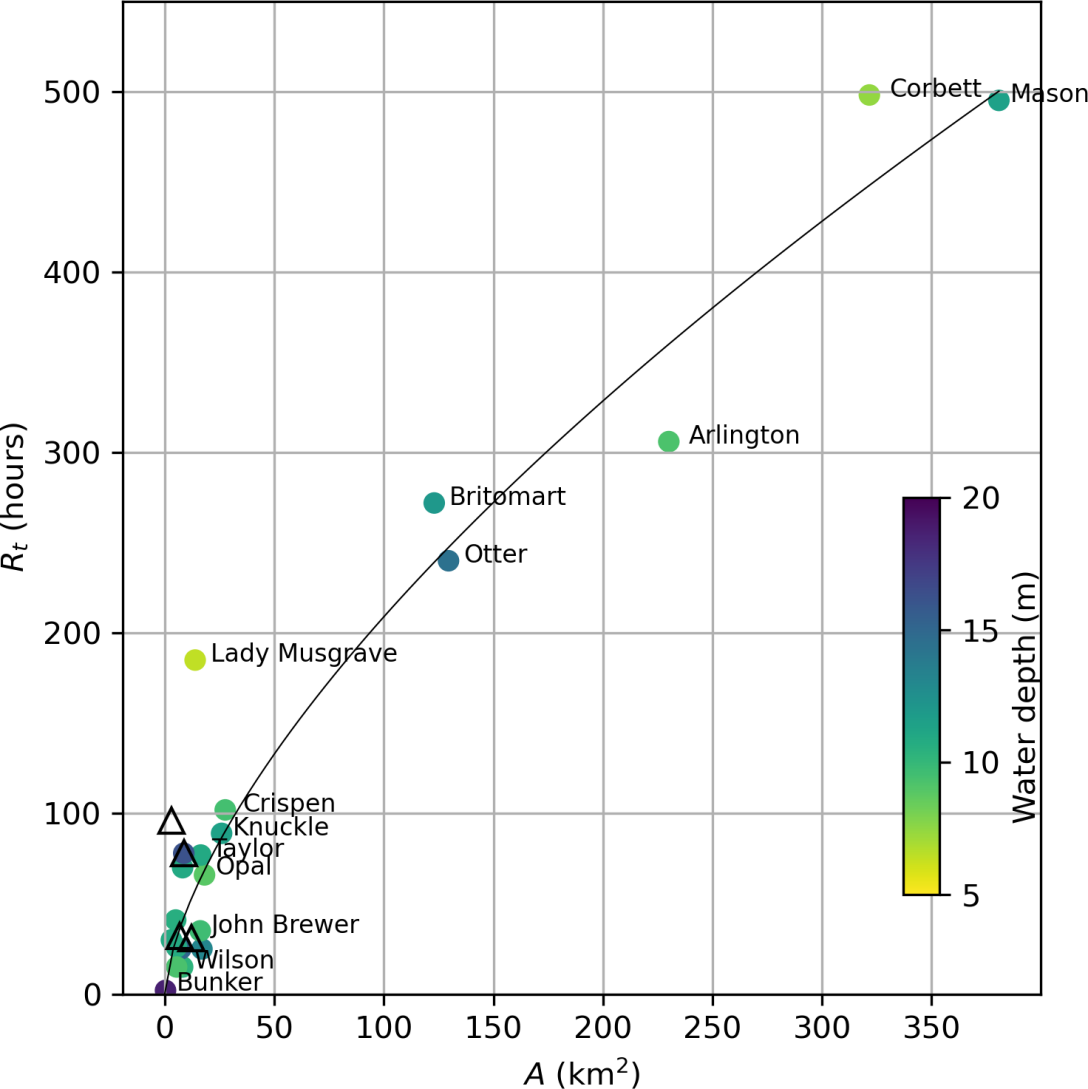
Time taken to reach 5.0% retention (*R_t_*, h) plotted against reef area (*A*, km^2^) for 20 submerged reefs (circles) and four island reefs (triangles). Additionally, for the submerged reefs, symbol fill colour represents mean water depth (*h*, m) inside of the 20 m depth contour. The line is a least-squares fitting (*r*^2^ = 0.91) of the function *R_t_* = 10.34(*A*)^0.65^ applied to all reefs.

Loss of particles from reefs (figure 6) and associated estimates of retention time vary widely across the 24 reefs studied. While there are many factors that may contribute to these differences (e.g. regional current strength and direction, tidal current strength, bathymetry, reef size), the relationship between retention time (*R_t_*) and reef area (*A*) shown in figure 8 suggests that most of the variation between reefs (*n* = 24) can be empirically explained by the following power law relationship (*r*^2^ = 0.91):


(3.1) $$R_{t}=10.34.A^{0.65}.$$

**Figure 9.**
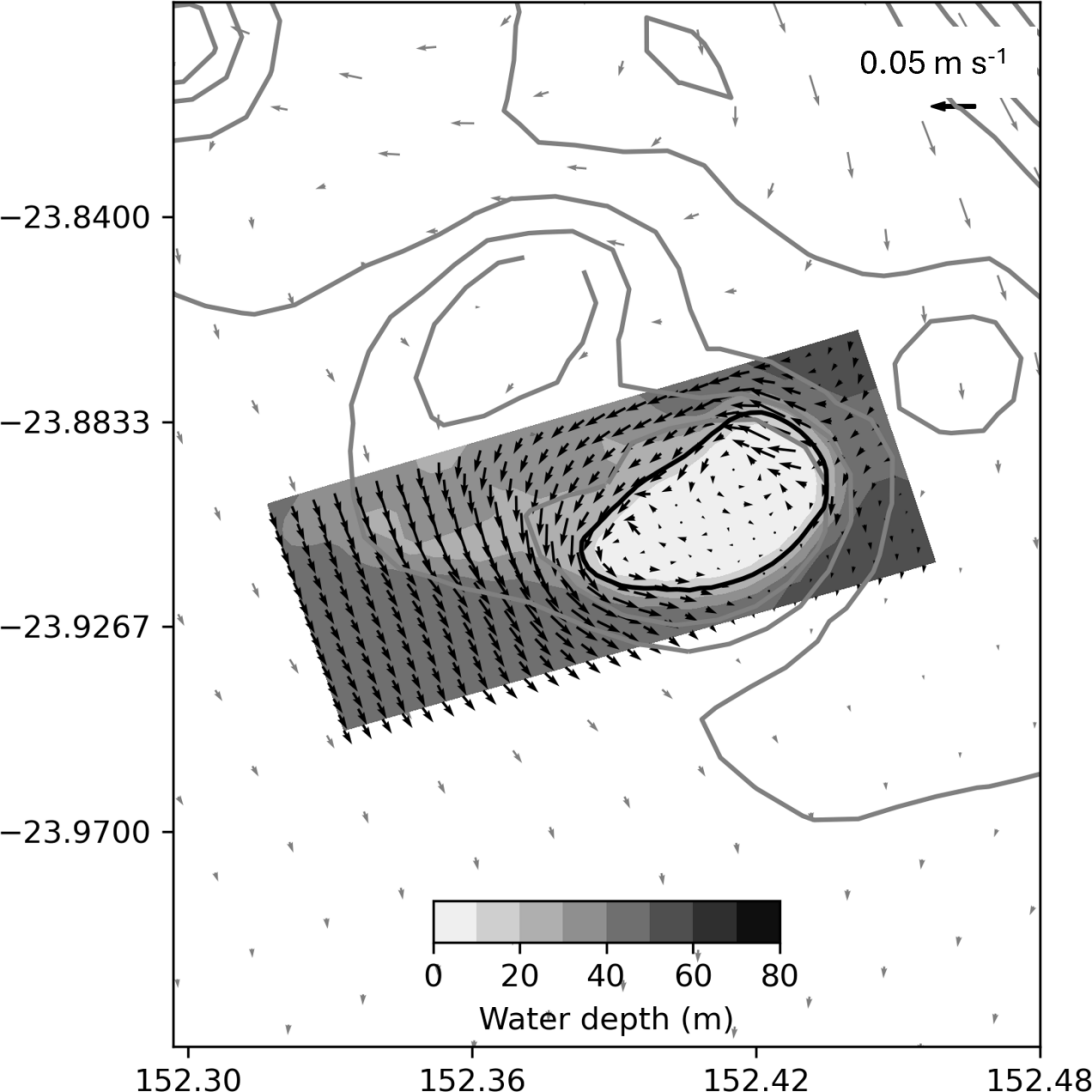
Depth-averaged mean flow (vector arrows) averaged between 20 and 25 December for Lady Musgrave reef in the parent (grey arrows) and nested (black arrows) model domain. Filled contours depict the bathymetry of the nested model according to the grey colour scale shown, and grey line contours show the bathymetry from the parent model at 10 m intervals starting at 30 m. The 20 m depth contour from the nested model bathymetry used in the numerical experiments to define reef area is also shown in black. Arrow density for both the parent and nested models has been reduced by a factor of two for clarity.

A notable outlier to this relationship is Lady Musgrave reef, where retention time is underestimated by more than 5 days. Lady Musgrave is a shallow reef located in the far southern GBR at 24.8° S (mean depth 6.4 m; electronic supplementary material, table S1) that tends to divert flow around its perimeter, limiting exchange with deeper water and resulting in low mean currents across the top of the reef, thus probably ‘trapping’ the particles (figure 9).

**Figure 10.**
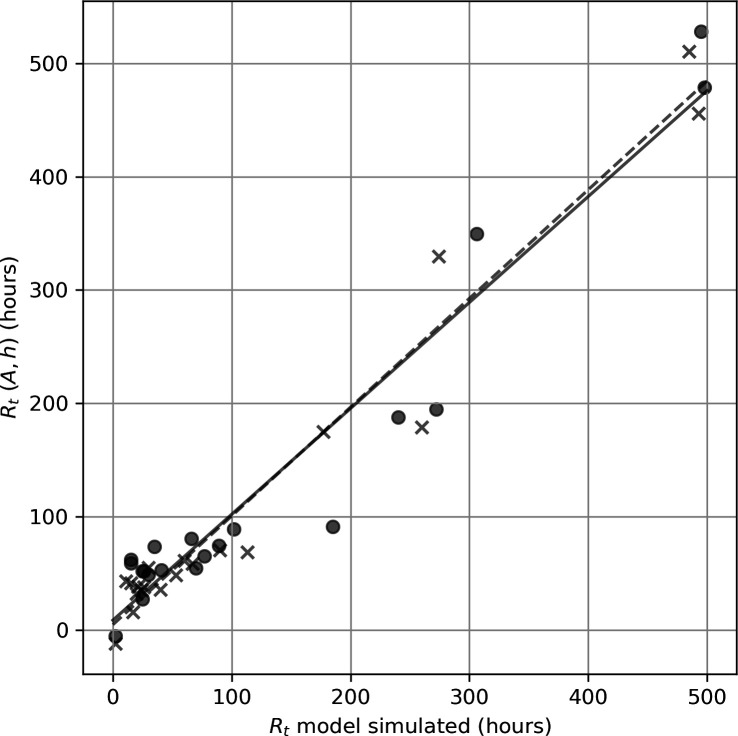
Time taken to reach 5% retention over the submerged reefs (circles and solid line) predicted by the multiple linear regression model *R_t_* = 1.23*A* − 6.38 h (*r^2^* = 0.92) where *A* is reef area (km^2^) and *h* is mean water depth (m) compared with retention time simulated by the particle-tracking model experiments. A similar linear relationship is obtained (*r^2^* = 0.95) when 10% retention is used to define the retention time (crosses and dashed line), noting that the dashed line is partly obscured by the solid line.

Multiple linear regression shows (figure 10) that a combination of reef area and mean water depth for the submerged reefs (*n* = 20) provides an alternative linear predictor (*r*^2^ = 0.92) of particle retention time estimated by the particle-tracking experiments of the form:


(3.2) $$R_{t}=1.23\left(A\right)-6.38\left(\overset{-}{h}\right).$$

This close linear relationship is maintained (*r*^2^ = 0.95) when 10% retention is used to define the retention time rather than 5% (see dashed line in figure 10).

**Figure 11.**
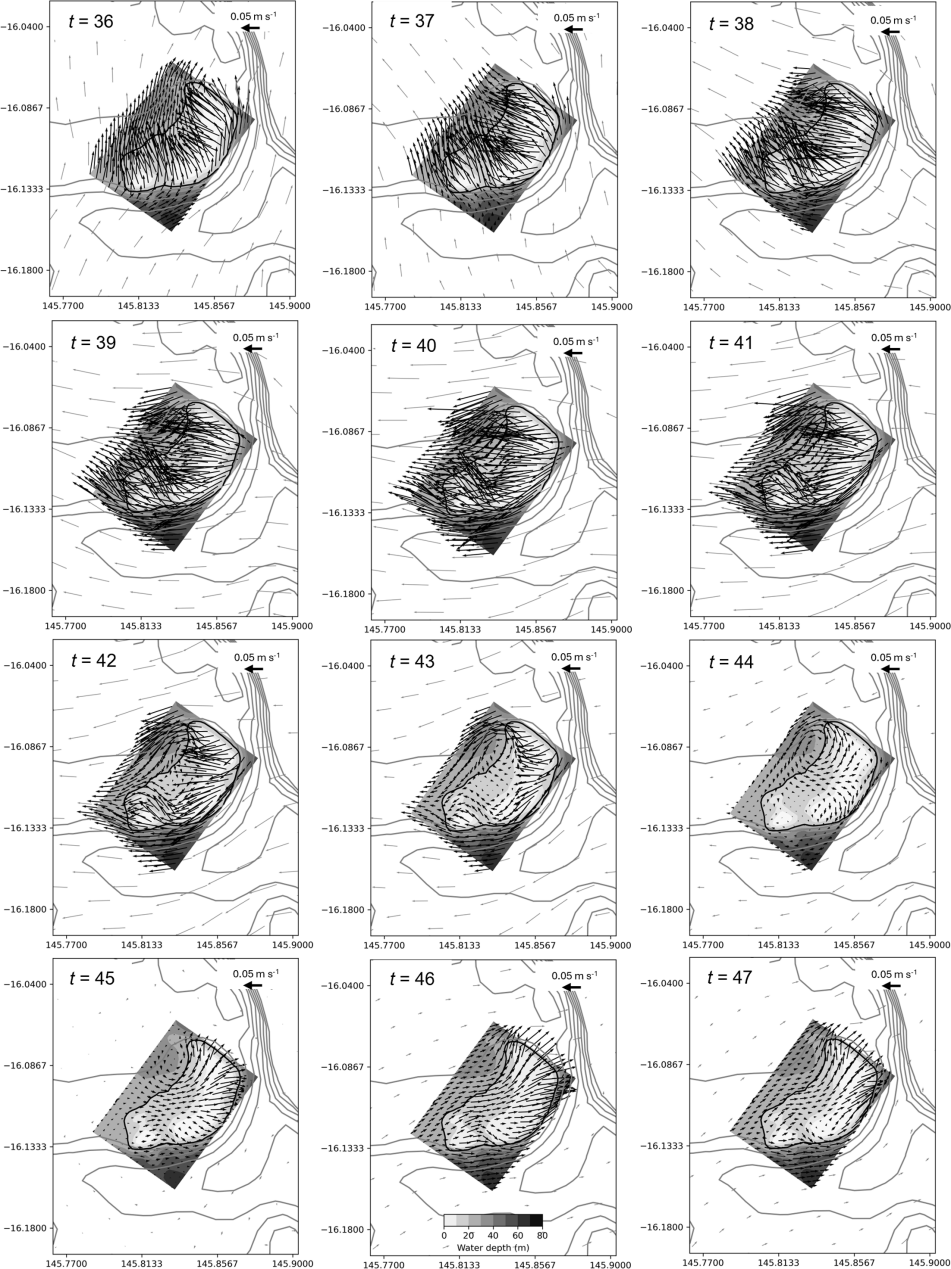
Hourly variation in the velocity field for Crispen reef over 12 h between *t* = 36 and *t* = 47 h (where *t* is the time in hours from the start of the particle simulation). Filled contours (shadings) depict bathymetry of the nested model according to the grey colour scale shown in the bottom central panel, and grey line contours show bathymetry from the parent model at 10 m intervals starting at 30 m. The 20 m depth contour from the nested model bathymetry used in the numerical experiments to define reef area is shown as a solid black line. Arrow density for both the parent and nested models has been reduced by a factor of two for clarity.

**Figure 12.**
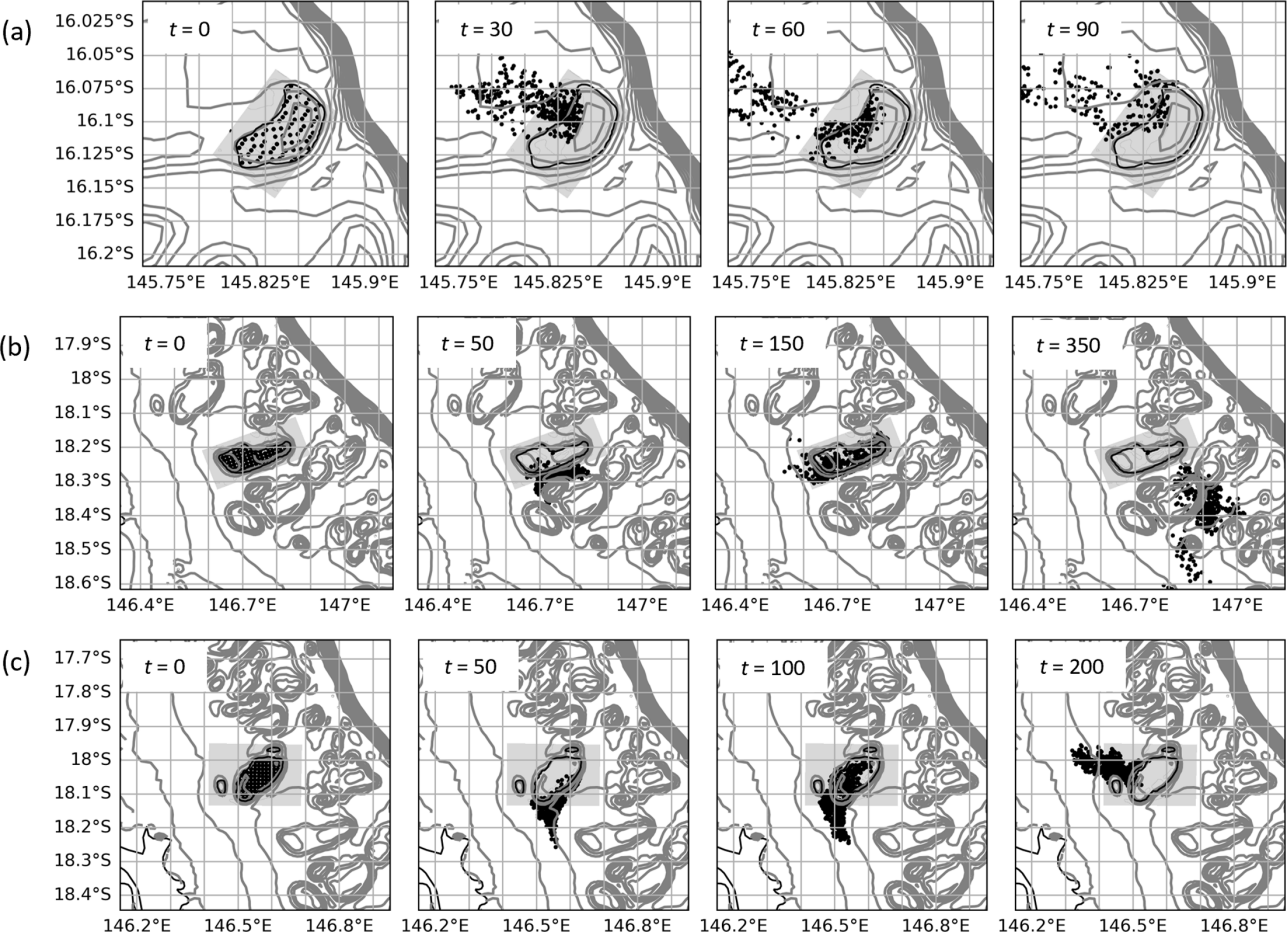
Particle distribution (black circles) at fixed time intervals, *t* (h), from midnight on 20 December 2016, during numerical dispersal experiments for (a) Crispen, (b) Britomart, and (c) Otter reefs. Grey lines depict bathymetric contours, and grey shading shows the domain of the fine-resolution nested grid. The reef perimeter is defined by the black 20 m contour line.

The reefs in the central and northern GBR with the longest estimated retention times, Crispen Otter, Britomart, Arlington, Corbett and Mason (figure 8), are all characterized by a recovery of particle numbers over the reef following an initial loss of at least 80% of particles to the surrounding waters (figure 6b,c). For the reefs in the central GBR, tidal eddies appear to play an important role in retaining larvae, as discovered by Black & Gay [55]. For example, closer examination of the hourly velocity field for Crispen reef during a 12 h period (36–47 h) when particle numbers are recovering over the reef (see figure 6b) reveals that an eddy starts to form on the western side of the reef at *t* = 43 h (figure 11), 1 h before the high tide. The eddy continues to strengthen as the free stream flow is stationary at *t* = 45. Similar features occur on each tidal cycle (although not always as distinct) on the western side of the reef. This corresponds closely with the area where particles tend to accumulate, keeping them adjacent to the reef edge for a prolonged period (figure 12a).

**Figure 13.**
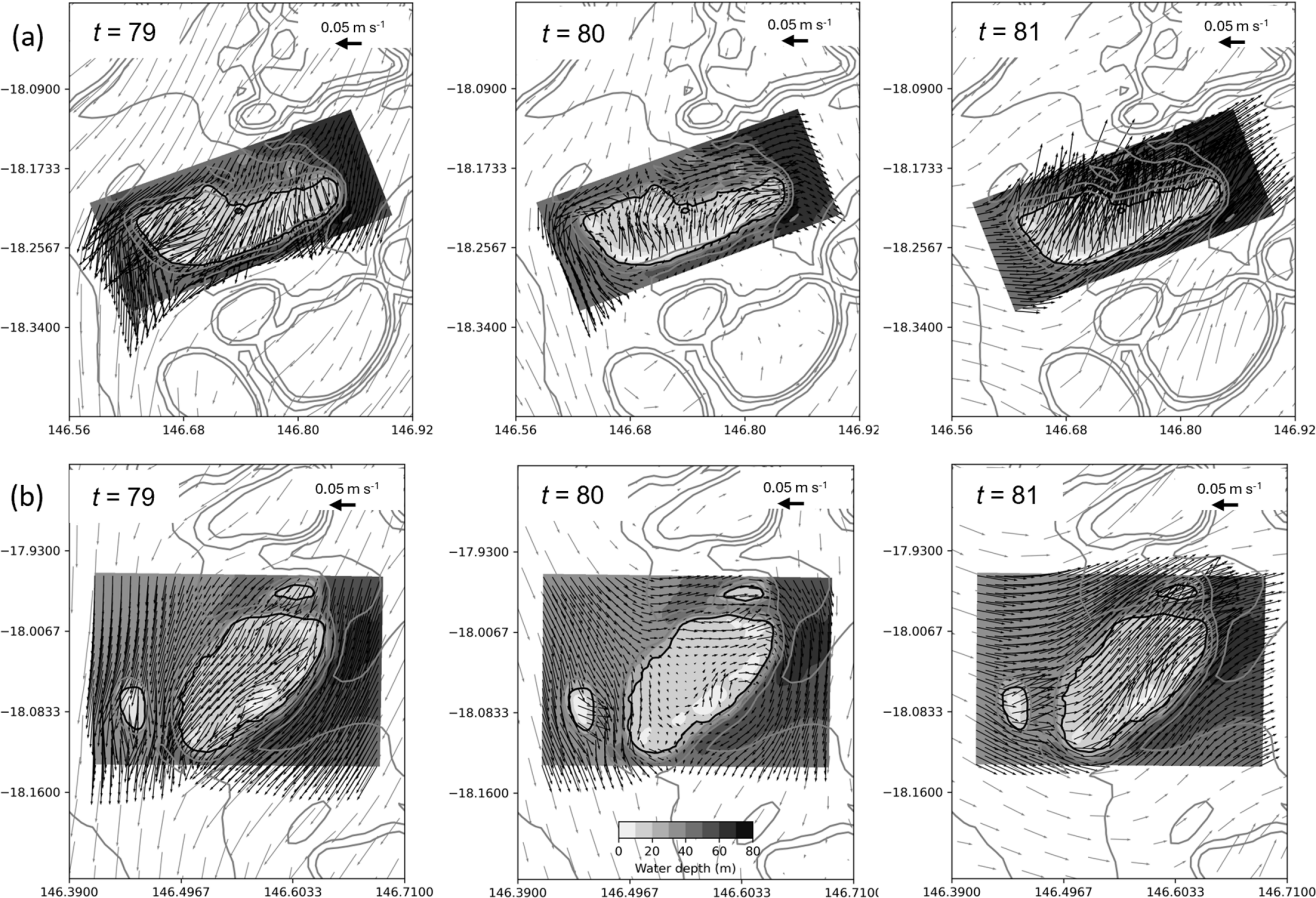
Variation in depth-averaged velocity field for (a) Britomart, and (b) Otter reefs, over a 3 h period centred on a high tide at 80 h from the start of the simulation. Filled contours depict bathymetry of the nested model according to the grey colour scale shown in the bottom central panel, and grey line contours show bathymetry from the parent model at 10 m intervals starting at 30 m. The 20 m depth contour from the nested model bathymetry used in the numerical experiments to define reef area is also shown in black. Arrow density for both the parent and nested models has been reduced by a factor of three for clarity.

Tidal eddies are also observed on the south-west corner of both Otter and Britomart reefs closely located together in the central GBR as the tide reaches its maximum height. Figure 13 shows an example of this for the high tide that occurs at 80 h when both reefs are simultaneously displaying a gradual recovery of particle numbers over the reef top (see figure 6c). As with Crispen reef, this corresponds with the region where higher particle densities are maintained (figure 12b,c). Similar re-circulation features are also observed at Arlington reef around high tide (not shown).

**Figure 14.**
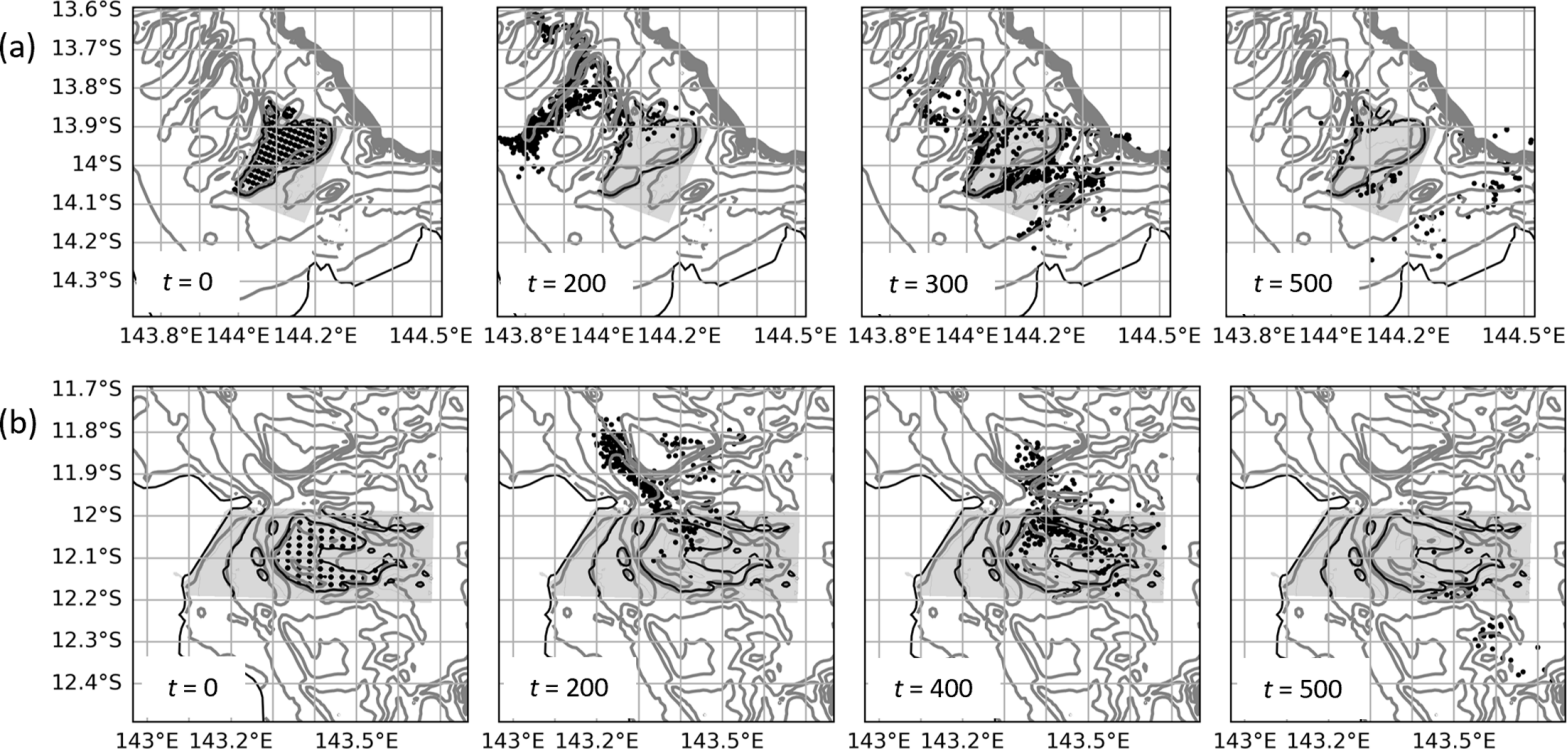
Particle distribution (black circles) at fixed time intervals, *t* (h), from midnight on 20 December 2016, during numerical dispersal experiments for (a) Corbett, (b) Mason reefs. Grey lines depict bathymetric contours, and grey shading shows the domain of the fine-resolution nested grid. The reef perimeter is defined by the black 20 m contour line.

At the largest reefs included in this study, Corbett and Mason in the northern GBR, a quite different process is taking place that returns particles back to the reef at longer time scales. These reefs are in areas of complex bathymetry including narrow reef channels that slow down dispersal, with the result that even after approximately 8 days (200 h) most particles have only moved a relatively short distance away (figure 14). Subsequently, when the dominant flow changes direction, many of the particles re-visit the reef two to three weeks after their initial release, resulting in the unusually long retention times estimated in this study.

In contrast to the submerged reefs, particle retention on the island reefs was found to be largely independent of area and water depth. For example, retention around Fitzroy Island is high (104 h) despite having a small reef area (3.02 km^2^) and deep water (mean depth 16.4 m). Having modelled only a small number of island reefs, we have been unable to find any reliable predictors of their retention time.

## 4. Discussion

Numerical modelling results presented here show that dispersal of passive particles from individual reefs of the GBR are strongly dependent on whether the particles are well mixed within the water column and dispersed, on average, by the depth-averaged velocity or restricted to the surface layer and dispersed by the surface velocity. Well-mixed particles typically experience slower net transport away from the reef (figure 3) owing to reduced wind influence and more circuitous trajectories with tidal reversals (figure 4). The relationship between surface and depth-averaged velocities varies with tidal phase and reef bathymetry (figure 5), making comparison between previous studies difficult. In fact, little is known about how the vertical distributions of coral larvae change over time, but it is axiomatic that at some point, after attaining competency, the larvae must arrive at the seabed to settle. In addition, the model simulations reported here (electronic supplementary material, figure S2) show that vertical turbulent mixing can be very strong and highly variable over shallow reefs, easily nullifying the buoyancy or vertical positioning by larvae. Vertical velocity can also be high around steep seabed topography [56] adding to the likelihood that any vertical stratification of larvae will be short-lived. Until better information becomes available on vertical distributions of coral larvae, depth-averaged velocities appear to provide the most reliable basis for two-dimensional connectivity modelling during the early phase of dispersal before larvae move into deeper stratified waters.

The enhancement of local retention of particles with grid resolution (figure 7) is consistent with the study of [27] who compared five models of differing grid resolution between 250 m and 4 km and found that particles stayed closer to their source (natal) reef for longer with finer resolution models. While model grid resolution is often restricted by computational resources, any reef management recommendations based on modelling results such as those reported here should only be made for scales that are well resolved by the modelling.

Overall, we have found that large-area submersed shallow-water reefs tend to have higher rates of local retention (figure 10) with reef size accounting for most of the variation (figure 8). This is broadly consistent with Black *et al*. [22] who found a trend of increased particle retention with reef area (with some exceptions) and is in part related to the increased distance that particles must travel to vacate larger-sized reefs. This probably explains the lower part of the curve in figure 8, making its gradient dependent on the magnitude of the currents, which are known to be highly variable on the GBR [57]. This is consistent with the modelling results of Cetina-Heredia & Connolly [58] which show mean water residence time for GBR reefs can be predicted from a function of flow speed, reef dimension and vertical diffusivity. By contrast, the upper part of the curve in figure 8 probably reflects two different processes associated with some of the larger reefs (Crispen, Otter, Britomart, Arlington, Corbett and Mason) where particles that had previously moved off the reef into deeper surrounding waters are recaptured, thereby greatly extending the overall retention time of the reef (figure 6b,c). Firstly, there is evidence from our model simulations that recycling of particles, for the larger reefs in the central GBR, is partly owing to tidal eddies forming in the lee of the reef as the tide reaches its maximum height (figures 11 and 13). Particles tend to accumulate in these eddy locations, keeping them close to the reef edge, where they are easily available to re-populate the reef following a change in flow direction (figure 12). A different effect is seen for the two largest reefs, Mason and Corbett, located in the northern GBR region, where complex bathymetry including narrow reef channels in the surrounding area dramatically slows down dispersal of particles to the extent that they can return to the reef up to two to three weeks later when the dominant flow changes direction. This is reminiscent of the dense reef matrix effect observed by Andutta *et al*. [20] for the Swains GBR region.

Topographically generated eddies related to both tidal and regional currents are known to be important physical mechanisms supporting retention of biological propagules and pre-settlement fishes close to submerged reefs and islands on a variety of spatial and temporal scales [21,58–60]. These features largely form from secondary three-dimensional circulation and will not be adequately captured by surface velocity fields, further favouring the choice of depth-averaged velocities for use in two-dimensional dispersal model experiments. It is also possible that the more prolonged recycling of particles as the reefs get bigger could be related to the length of the residual circulation in the lee of the reef, which is known to be dependent on the reef cross section perpendicular to the current [28] . However, the nested model domains used in the present study may be too restricted to adequately resolve these features and fully capture the reef’s zone of influence. Further modelling work is needed with an improved high-resolution model set-up that covers larger areas of the Great Barrier Reef.

It is worth noting that this type of effect is implicit in the analytical model of Black *et al*. [22] because their reef area definition included the area of re-circulation in the lee of the reef, as well as the distance covered by the tidal excursion. Including these regions of recirculating water in the definition of reef area explains why Black *et al*. [22] obtain monotonic exponential decay of particles largely free of the oscillations observed here and report relatively long residence times.

The type of relationships established here relating retention on submerged reefs to physical parameters like reef area and mean water depth (equations (3.1) and (3.2)) have the potential to be incorporated into larval connectivity models and reef meta-population models by combining them with information on larval competency [25] and post-settlement mortality [33] to estimate local coral recruitment rates. In this case, local recruitment could be scaled for reefs of differing sizes. We note that empirical relationships reflect the net effect of a combination of processes; therefore, the estimated retention times themselves should be used with caution pending further research given the limitations we have discussed. It is also worth noting that the retention time of different reefs is estimated here by using an arbitrary threshold of 5% or 10% of the total particle population for the purpose of comparing the reefs. While these two thresholds give similar results (figure 10), higher thresholds are more complex because of the often non-monotonic decay of particle concentration (figure 6). This contrasts with previous studies that report approximately exponential decay of particle numbers, allowing them to use non-threshold metrics to compare retention between reefs, such as the *e*-folding time (the time interval in which an exponentially changing quantity reduces by a factor of *e* = 2.71828) [20] or the exponential rate constant [22].

In this study, we have taken advantage of previous modelling work [52] to examine dispersal patterns from a range of individual reefs along the GBR following a single coral spawning event in December 2016. More work is needed to understand how this might vary in different years. This could be especially important for the re-circulation of particles observed for the larger submerged reefs. This study is also restricted by our seabed-depth definition of reef area. This could be problematic for reefs fringing a large land mass. Nevertheless, this definition works well for mid-shelf and offshore reefs of the GBR and some other reef systems globally where shallow reefs are surrounded by deeper water. It is also relevant for assessing retention time relationships in GBR research and management. The GBR Marine Park Authority (GBRMPA) zoning typically identifies GBR reef systems of approximately the same size as our seabed-depth definition (e.g. Arlington reef is one zone). Furthermore, ecological studies of the GBR such as that of Bozec *et al*. [29] and [31,32] use networks of reefs linked by connectivity matrices, where the connection is between reefs identified by the same GBRMPA zoning, and retention within individual reefs is an important model parameter. Overall, our definition of reef area is designed to match both ecological processes and practical modelling and management investigations.

Island reefs are complicated by the fact that the land mass obstructs dispersal, and they require special attention. There is some evidence that this obstruction effect is enhanced for larger islands like Fitzroy compared with smaller ones like Double Cone and Low Wood, but this is inconclusive. An added complication is met at Lizard Island where particles must navigate three separate land masses. In any case, the addition of artificial velocities around the islands in the present study to prevent particles getting stranded modifies the way in which particles move around the perimeter of the island making any deductions doubtful, and this problem will require further work.

## Data Availability

This work uses hydrodynamic model output at two different grid resolutions that is freely available via the two following persistent links: course resolution model (referred to as 'parent mode' in the manuscript) https://thredds.nci.org.au/thredds/catalog/fx3/gbr1_2.0/catalog.html; and fine resolution model (referred to as 'reef models' in the manuscript) [61]. Supplementary material is available online [62].

## References

[1] Eddy TD, Lam VWY, Reygondeau G, Cisneros-Montemayor AM, Greer K, Palomares MLD, Bruno JF, Ota Y, Cheung WWL. 2021 Global decline in capacity of coral reefs to provide ecosystem services. One Earth **4**, 1278–1285. (10.1016/j.oneear.2021.08.016)

[2] Gouezo M, Wolanski E, Critchell K, Fabricius K, Harrison P, Golbuu Y, Doropoulos C. 2021 Modelled larval supply predicts coral population recovery potential following disturbance. Mar. Ecol. Prog. Ser. **661**, 127–145. (10.3354/meps13608)

[3] Mumby PJ, Mason RAB, Hock K. 2021 Reconnecting reef recovery in a world of coral bleaching. Limnol. Oceanogr. **19**, 702–713. (10.1002/lom3.10455)

[4] Feng M, Colberg F, Slawinski D, Berry O, Babcock R. 2016 Ocean Circulation drives heterogenous recruitments and connectivity among coral populations on the North West Shelf of Australia. J. Mar. Syst. **164**, 1–12.

[5] Hock K, Wolff NH, Condie SA, Anthony KRN, Mumby PJ. 2014 Connectivity networks reveal the risks of crown‐of‐thorns starfish outbreaks on the Great Barrier Reef. Journal of Applied Ecology **51**, 1188–1196. (10.1111/1365-2664.12320)

[6] Cowen RK, Sponaugle S. 2009 Larval dispersal and marine population connectivity. Ann. Rev. Mar. Sci. **1**, 443–466. (10.1146/annurev.marine.010908.163757) 21141044

[7] Boschetti F, Babcock RC, Doropoulos C, Thomson DP, Feng M, Slawinski D, Berry O, Vanderklift MA. 2019 Setting priorities for conservation at the interface between ocean circulation, connectivity, and population dynamics. Ecol. Appl. **30**, 1. (10.1002/eap.2011) 31556209

[8] Doropoulos C, Babcock RC. 2018 Harnessing connectivity to facilitate coral restoration. Front. Ecol. Environ. **16**, 558–559. (10.1002/fee.1975)

[9] Harrison HB *et al*. 2012 Laval export from marine reserves and the recruitment benefit for fish and fisheries. Curr. Biol. **22**, 1023–1028. (10.1016/j.cub.2012.04.008) 22633811

[10] Game ET, McDonald-Madden E, Puotinen ML, Possingham HP. 2008 Should we protect the strong or the weak? Risk, resilience, and the selection of Marine Protected Areas. Conserv. Biol. **22**, 1619–1629. (10.1111/j.1523-1739.2008.01037) 18759769

[11] Figueiredo J, Thomas CJ, Deleersnijder E, Lambrechts J, Baird AH, Connolly SR, Hanert E. 2022 Global warming decreases connectivity among coral populations. Nat. Clim. Chang. **12**, 83–87. (10.1038/s41558-021-01248-7)

[12] Saavendra-Sotelo NC, Calderon-Aguilera LE, Reyes-Bonilla H, López-Pérez RA, Medina-Rosas P, Rocha-Olivares A. 2011 Limited genetic connectivity of Pavona gigantea in the Mexican Pacific. Coral Reefs **30**, 677–686. (10.1007/s00338-011-0742-6)

[13] Ayre DJ, Hughes TP. 2000 Genotypic diversity and gene flow in brooding and spawning corals along the Great Barrier Reef, Australia. Evolution **54**, 1590–1605. 11108587 10.1111/j.0014-3820.2000.tb00704.x

[14] Gilmour JP, Smith LD, Brinkman RM. 2009 Biannual spawning, rapid larval development and evidence of self-seeding for scleractinian corals at an isolated system of reefs. Mar. Biol. **156**, 1297–1309. (10.1007/s00227-009-1171-8)

[15] Sammarco PW, Andrews JC. 1988 Localized dispersal and recruitment in Great Barrier-Reef corals – the helix experiment. Science **239**, 1422–1424. (10.1126/science.239.4846.1422) 17769738

[16] Hata T, Madin JS, Cumbo VR, Denny M, Figueiredo J, Harii S, Thomas CJ, Baird AH. 2017 Coral larvae are poor swimmers and require fine-scale reef structure to settle. Sci. Rep. **7**, 2249. (10.1038/s41598-017-02402-y) 28533550 PMC5440398

[17] Raimondi PT, Morse ANC. 2000 The consequences of complex larval behavior in a coral. Ecology **81**, 3193–3211. (10.1890/0012-9658(2000)081[3193:tcoclb]2.0.co;2)

[18] Sammarco PW, Andrews JC. 1989 The Helix experiment: differential localized dispersal and recruitment patterns in great barrier reef corals. Limnol. Oceanogr. **34**, 896–912. (10.4319/lo.1989.34.5.0896) 17769738

[19] Condie S, Condie R. 2016 Retention of plankton within ocean eddies. Glob. Ecol. Biogeogr. **25**, 1264–1277. (10.1111/geb.12485)

[20] Andutta FP, Kingsford MJ, Wolanski E. 2012 ‘Sticky water’ enables the retention of larvae in a reef mosaic. Estuar. Coast. Shelf Sci. **101**, 54–63. (10.1016/j.ecss.2012.02.013)

[21] Burgess S, Kingsford M, Black K. 2007 Influence of tidal eddies and wind on the distribution of presettlement fishes around One Tree Island, Great Barrier Reef. Mar. Ecol. Prog. Ser. **341**, 233–242. (10.3354/meps341233)

[22] Black KP, Gay SL, Andrews JC. 1990 Residence times of neutrally-buoyant matter such as larvae, sewage or nutrients on coral reefs. Coral Reefs **9**, 105–114. (10.1007/bf00258221)

[23] Aoki N, Weiss B, Jézéquel Y, Zhang WG, Apprill A, Mooney TA. 2024 Soundscape enrichment increases larval settlement rates for the brooding coral Porites astreoides. R. Soc. Open Sci. **11**, 231514. (10.1098/rsos.231514) 38481984 PMC10933538

[24] Gleason DF, Hofmann DK. 2011 Coral larvae: from gametes to recruits. J. Exp. Mar. Biol. Ecol. **408**, 42–57. (10.1016/j.jembe.2011.07.025)

[25] Randall CJ, Giuliano C, Stephenson B, Whitman TN, Page CA, Treml EA, Logan M, Negri AP. 2024 Larval precompetency and settlement behaviour in 25 Indo-Pacific coral species. Commun. Biol. **7**, 142. (10.1038/s42003-024-05824-3) 38297134 PMC10830509

[26] Figueiredo J, Baird AH, Connolly SR. 2013 Synthesizing larval competence dynamics and reef-scale retention reveals a high potential for self-recruitment in corals. Ecology **94**, 650–659. (10.1890/12-0767.1) 23687891

[27] Saint-Amand A, Lambrechts J, Hanert E. 2023 Biophysical models resolution affects coral connectivity estimates. Sci. Rep. **13**, 9414. (10.1038/s41598-023-36158-5) 37296146 PMC10256739

[28] Wolanski E, Imberger J, Heron ML. 1984 Island wakes in shallow coastal waters. J. Geophys. Res. **89**, 10553–10569. (10.1029/JC089iC06p10553)

[29] Bozec Y, Hock K, Mason RAB, Baird ME, Castro‐Sanguino C, Condie SA, Puotinen M, Thompson A, Mumby PJ. 2022 Cumulative impacts across Australia’s Great Barrier Reef: a mechanistic evaluation. Ecol. Monogr. **92**, 1494. (10.1002/ecm.1494)

[30] Dorman JG, Castruccio FS, Curchitser EN, Kleypas JA, Powell TM. 2016 Modeled connectivity of Acropora millepora populations from reefs of the Spratly Islands and the greater South China Sea. Coral Reefs **35**, 169–179. (10.1007/s00338-015-1354-3)

[31] Condie SA *et al*. 2021 Large-scale interventions may delay decline of the Great Barrier Reef. R. Soc. Open Sci **8**, 201296. (10.1098/rsos.201296) 34007456 PMC8080001

[32] Condie SA, Plagányi ÉE, Morello EB, Hock K, Beeden R. 2018 Great Barrier Reef recovery through multiple interventions. Conserv. Biol. **32**, 1356–1367. (10.1111/cobi.13161) 29956854

[33] Connolly SR, Baird AH. 2010 Estimating dispersal potential for marine larvae: dynamic models applied to scleractinian corals. Ecology **91**, 3572–3583. (10.1890/10-0143.1) 21302829

[34] Arai T, Kato M, Heyward A, Ikeda T, Iizuka T, Maruyama T. 1993 Lipid composition of positively buoyant eggs of reef building corals. Coral Reefs **12**, 71–75.

[35] Oliver JK, Willis BL. 1987 Coral-spawn slicks in the Great Barrier Reef: preliminary observations. Mar. Biol. **94**, 521–529.

[36] Harii S, Nadaoka K, Yamamoto M, Iwao K. 2007 Temporal changes in settlement, lipid content and lipid composition of larvae of the spawning hermatypic coral Acropora tenuis. Mar. Ecol. Prog. Ser. **346**, 89–96. (10.3354/meps07114)

[37] Tay YC, Guest JR, Chou LM, Todd PA. 2011 Vertical distribution and settlement competencies in broadcast spawning coral larvae: Implications for dispersal models. J. Exp. Mar. Biol. Ecol. **409**, 324–330.

[38] Mulla AJ, Lin CH, Takahashi S, Nozawa Y. 2021 Photo-movement of coral larvae influences vertical positioning in the ocean. Coral Reefs **40**, 1297–1306. (10.1007/s00338-021-02141-7)

[39] Reidenbach MA, Monismith SG, Koseff JR, Yahel G, Genin A. 2006 Boundary layer turbulence and flow structure over a fringing coral reef. Limnol. Oceanogr. **51**, 1956–1968.

[40] Condie SA. 1999 Settling regimes for non-motile particles in stratified waters. Deep Sea Res. I **46**, 681–699. (10.1016/s0967-0637(98)00085-5)

[41] Smith CL, Hill AE, Foreman MGG, Peña MA. 2001 Horizontal transport of marine organisms resulting from interactions between diel vertical migration and tidal currents off the west coast of Vancouver Island. Can. J. Fish. Aquat. Sci. **58**, 736–748. (10.1139/cjfas-58-4-736)

[42] Ani CJ, Haller-Bull V, Gilmour JP, Robson BJ. 2024 Connectivity modelling identifies sources and sinks of coral recruitment within reef clusters. Sci. Rep. **14**, 13564. (10.1038/s41598-024-64388-8) 38866879 PMC11169499

[43] Takeyasu K, Uchiyama Y, Mitarai S. 2023 Quantifying connectivity between mesophotic and shallow coral larvae in Okinawa Island, Japan: a quadruple nested high-resolution modeling study. Front. Mar. Sci. **10**, 1174940. (10.3389/fmars.2023.1174940)

[44] Vogt-Vincent NS, Mitarai S, Johnson HL. 2023 High-frequency variability dominates potential connectivity between remote coral reefs. Limnol. Oceanogr. **68**, 2733–2748.

[45] Hock K, Doropoulos C, Gorton R, Condie SA, Mumby PJ. 2019 Split spawning increases robustness of coral larval supply and inter-reef connectivity. Nat. Commun. **10**. (10.1038/s41467-019-11367-7) PMC6671964 31371712

[46] Delandmeter P, van Sebille E. 2019 The parcels v2.0 Lagrangian framework: new field interpolation schemes. Geosci. Model Dev. **12**, 3571–3584. (10.5194/gmd-12-3571-2019)

[47] Steven ADL, Baird ME, Brinkman R, Car NJ, Cox SJ, Herzfeld M. 2019 An operational information system for managing the Great Barrier Reef: eReefs. J. Oper. Ocean. **12**, S12–S28. (10.1080/1755876X.2019.1650589)

[48] Schiller A, Herzfeld M, Brinkman R, Stuart G. 2014 Monitoring, predicting and managing one of the seven natural wonders of the world. Bull. Am. Meteorol. Soc. **95**, 23–30. (10.1175/BAMS-D-12-00202.1)

[49] Herzfeld M. 2009 Improving stability of regional numerical ocean models. Ocean Dyn. **59**, 21–46. (10.1007/s10236-008-0158-1)

[50] Herzfeld M, Gillibrand PA. 2015 Active open boundary forcing using dual relaxation time-scales in downscaled ocean models. Ocean Model. **89**, 71–83. (10.1016/j.ocemod.2015.02.004)

[51] Baird ME, Green R, Lowe R, Mongin M, Bougeot E. 2020 Optimising cool-water injections to reduce thermal stress on coral reefs of the Great Barrier Reef. PLoS ONE **15**, e0239978. (10.1371/journal.pone.0239978) 33079939 PMC7575073

[52] Baird ME, Mongin M, Rizwi F, Bay LK, Cantin NE, Morris LA, Skerratt J. 2021 The effect of natural and anthropogenic nutrient and sediment loads on coral oxidative stress on runoff-exposed reefs. Mar. Pollut. Bull. **168**, 112409. (10.1016/j.marpolbul.2021.112409) 33957497

[53] Babcock RC, Bull GD, Harrison PL, Heyward AJ, Oliver JK, Wallace CC, Willis BL. 1986 Synchronous spawnings of 105 Scleractinian coral species on the Great-Barrier-Reef. Mar. Biol. **90**, 379–394. (10.1007/BF00428562)

[54] Kaandorp MLA, Dijkstra HA, van Sebille E. 2020 Closing the Mediterranean marine floating plastic mass budget: inverse modeling of sources and sinks. Environ. Sci. Technol. **54**, 11980–11989. (10.1021/acs.est.0c01984) 32852202 PMC7547878

[55] Black KP, Gay SL. 1987 Eddy formation in unsteady flows. J. Geophys. Res. **92**, 9514–9522. (10.1029/jc092ic09p09514)

[56] Teague WJ, Wijesekera HW, Wang DW, Hallock ZR. 2022 Current observations on and around a deep-ocean island/reef: northern Palau and Velasco Reef. J. Oceanogr. **78**, 425–447. (10.1007/s10872-022-00647-4)

[57] Burrage DM, Black KP, Ness KF. 1994 Long-term current prediction in the central Great Barrier Reef. Cont. Shelf Res. **14**, 803–829. (10.1016/0278-4343(94)90074-4)

[58] Cetina-Heredia P, Connolly SR. 2011 A simple approximation for larval retention around reefs. Coral Reefs **30**, 593–605. (10.1007/s00338-011-0749-z)

[59] Black K, Moran P, Hammond L. 1991 Numerical models show coral reefs can be self-seeding. Mar. Ecol. Prog. Ser. **74**, 1–11. (10.3354/meps074001)

[60] Black KP. 1988 The relationship of reef hydrodynamics to variations in numbers of planktonic larvae on and around coral reefs. In Proc. 6th Int. Coral Reef Symp, 8-12 August 1988,Townsville, Australia, vol. **2**, pp. 125–130, Townsville, Australia: 6th International Coral Reef Symposium Executive Committee.

[61] CSIRO. 2024 Hydrodynamic model output for reefs of the great Barrier Reef. v1. CSIRO. Data Collection (10.25919/5mqs-5e27)

[62] Greenwood J, Sun CJ, Doropoulos C, Thomson D, Baird M, Porobic J *et al*. 2025 Supplementary material from: Passive retention of simulated larvae on coral reefs. Fighare. (10.6084/m9.figshare.c.7834011) PMC12105734 40421050

